# Reply to ‘Percutaneous coronary intervention timing and coronary physiology in transcatheter aortic valve implantation patients’

**DOI:** 10.1007/s12471-024-01861-z

**Published:** 2024-03-06

**Authors:** Hugo M. Aarts, Michiel Voskuil, Ronak Delewi

**Affiliations:** 1 Department of Cardiology, University Medical Centre Utrecht, Utrecht, The Netherlands; 2 Department of Cardiology, Amsterdam University Medical Centre, Amsterdam, The Netherlands

Dear Editor,

We thank Dr. Minten and colleagues for their interest and thorough review of our manuscript on the importance of preprocedural revascularisation in patients undergoing transcatheter aortic valve implantation (TAVI) [1]. The authors emphasise that further research is warranted to elucidate the role of percutaneous coronary intervention (PCI) in these patients, including the ideal timing of PCI, coronary physiology and the possible benefit regarding long-term outcomes.

Indeed, the observational design of the included studies in the current meta-analysis has its well-known inherent limitations, including selection bias. Fortunately, results from multiple well-organised randomised controlled trials are expected in the near future. Both the Dutch PRO-TAVI trial and the NOTION-3 trial are aiming to elucidate the benefit of PCI in patients with untreated significant coronary artery disease undergoing TAVI, including 5‑year clinical follow-up. Nonetheless, short-term clinical outcomes are also crucial in TAVI, as periprocedural complications are associated with increased mortality risk [2].

We believe that the results of these randomised controlled trials will contribute to a tailored-made therapy for patients with significant coronary artery disease undergoing TAVI.
